# Doctors Born, Not Made

**Published:** 1882-07

**Authors:** Ausburn Towner


### DOCTORS BORN, NOT MADE.

AUSBURN TOWNER.

Proverbs have been accurately termed "the wisdom of many and the wit of one." Short, sharp sentences of three or four words sometimes will encompass an amount of truth that it might take volumes to illustrate and develop.

The common expression: "When doctors disagree who shall decide," has a world of meaning in it. Who, indeed, can decide when learned men, or supposed learned men, dispute? Seeing such disagreements constantly, you are inclined to believe that their learning is all a matter of supposition at the best, a matter of opinion or guess-work, for it is not an easy matter to dispute about a fact or certainty. This is shown by the trite illustrations of the circulation of the blood and vaccination. Doctors agree now generally upon these matters, for experience, observation and fact have decided for them; but when the notions were first promulgated the fight over them in the profession was bitter and long. It should seem by this time, in what is called the great advance in the knowledge of anatomy, that opportunity on the part of doctors to disagree or dispute, would have been passed, as it has in the two instances named. That, given a certain state of affairs, certain other things done, certain results would follow always the same, like a proposition in logic or an example in any of the exact sciences. But this is not so. Cases are constantly arising in which the skill of the doctor is set entirely at naught; where his experience furnishes him no guide by which to go, and where whatever he may do, bring to him no such results as he expects, he becomes like a blind man in the dark, groping around in an unknown locality and apparently as ignorant as were his predecessors in the profession before the time of Hippocrates.

It might be observed here that the mysteries into which the ancient priests and physicians were inducted, and which seemed so solemn and awful to the common people, were so simply because the priests and physicians knew just as little as the common people themselves, but hid their own ignorance by a veil they themselves could not raise, and further, do not the successors of these wise men sometimes hide their own incertitude and want of knowledge in an owl-like demeanor that is very impressive and overpowering to the little-thinking?

It is not in what might be called the outer anatomical works of the human system does this seeming ignorance exist in the profession. These have been obtained, taken and accurately mapped out, so that in surgical operations an exactness has been obtained that can be relied upon. But the vital parts of the body, the citadel of the structure, has resisted, still resists and promises always to resist the attack that human intelligence has made upon it. You may say, and with truth, that anatomists have thoroughly dissected the human body; know where the minutest ligament, vein, muscle or cord belongs; how and why they act, and what are their functions. Still the supreme secret remains undisclosed. Here are paints, canvas, brushes, an easel and pallette. You know how they are used, but where is the picture the artist can produce? Here are pens, ink and paper. These you know also how to use. But where is the book, or poem that will stir the world? You can never answer these questions. Can the other ever be solved or understood? Toward the real secret of health and life it would seem we are not much farther advanced than were the so-called learned men of the time of Moses and Homer, who dissected living malefactors to discover, if possible, the secret springs of life.

An allusion to the case of the late President Garfield, so frequently named, may be forgiven, as it substantiates these things and is a very conspicuous instance. Alas! it is not alone. The station occupied by the suffering invalid has drawn public attention to the fact that has always existed; the uncertain tenure, not to call it by a harsher term, of our hold upon a knowledge of the construction of the human body and the functions of its numerous parts.

I say the case is not alone. It is a safe thing to assume, for it has been within the observation of the writer in enough instances to establish the truth of the assertion, that not one physician in the land, no matter what may be his experience, reputation or standing in the profession, who has not seen cases that have completely baffled him, or results, the causes for which it has been impossible for him to determine. Consultations being held would not settle the case, for there might be as many different opinions as there were doctors, and none of them might be right.

I can see but three solutions of these disagreements. Either the professed information and knowledge is all a farce, which would hardly be a fair, just or proper thing to say; or the doctors are imposters, who, granted that the knowledge is accurate, knew nothing of it; or granted still, that the knowledge is based on truth, there are a multitude of influences constantly at work modifying disease and sometimes changing entirely the relations to each other of the different parts of the human system. In other words, that there are certain broad rules which have been ascertained to be immutable, but the application of them is as various as there is variety in the human frame.

As, for instance, there are certain rules concerning the human face that are very rarely transgressed. Two eyes, a nose, mouth, cheek and forehead, with other features go always to make it up. Yet in that great variety Nature can and does dispose of these simple materials. Amidst the millions and millions of people of the world, no two faces are precisely alike, that some one cannot tell it from all others. Besides the mere mechanical arrangement too, there is another indefinable belonging to each one, separate and distinct from all others, the expression.

To know one face in all the world, is by no means to know all the faces in existence. To know one disease or the cast-iron rule for treating it, is by no means to know all, and yet all bodies are made alike mechanically, as are all faces.

It seems to me reasonable to suppose that the disagreements or surprises in the medical practice arise not from the want of knowledge or study, but from the too strict adherence to what that knowledge and study sets forth, from too much knowledge, it might be called. The books and the experience of others set forth that such a state of affairs should exist and it therefore must be so, when the fact is that there were never two sets of incidents and results all taken together that were precisely similar, any more than there are two faces in the world exactly alike.

In proportion as a physician is liberal-minded, not hide-bound by rules, and constantly alive to all that is new, in just such proportion will he be successful in his profession. He is the one who is never surprised, and who, in times of danger, so thoroughly sets forth his views and his reasons therefor, that all those who are associated with him must agree with him, if not at first, finally. It is to such men that the world and the profession owe all that it is. Such were Hippocrates and Galen. Such the great, pious and learned Boerhaase, whose fame was so extensive that his name was well-known in the then remote and little known region of China; such the famous and gifted, if rude, Abernethy of England, whose cases were some of the most marvellous ever performed; not to mention Harvey and many of our own time, learned, distinguished and successful.

It follows, therefore, that a physician, to fully merit the title of healer, must possess something else than a mere knowledge of the human frame and its various parts, and their relation to each other. It is something that he can never learn. It may be developed, enlarged and strengthened, for it was born with him when he was born, as much as is the poetic faculty given to a poet, or as used to be thought, a minister is called to preach the word of God. How many qualities must a skillful surgeon possess besides an accurate knowledge of anatomy? An eye that never errs, a quickness that rivals that of the lightning; a steadiness of nerve that nothing can disturb; a readiness that amounts to genius, and a mechanical precision almost microscopic! What a more powerful and more immaterial innate sense must a physician possess, who cannot see with his physical eye, but must judge solely from effects and appearances.

Of these are those who bestow good on their fellow-kind, whose services are in constant demand, and who, while winning a competence for themselves, earn the grateful appreciation of their fellow-creatures.

What should be said, on the other hand, of another class of men who enter the medical profession, not being inevitably drawn into it, but because it may give them a means of support, a high social position, or those upon whom they are dependent desire it? Of these are those who look not beyond the rules of the schools; who bring untold misery into the world; who follow a science round and round in one net, and if death results it is the fault of the patient and not of the system to which they adhere; and whose actions give rise to such proverbs as we set out with. Than to one of these, who would not trust himself rather to another who had manifested a "healing sense," although that other one might not know the thigh-bone from a shoulder-blade or might imagine that the diaphragm was a membrane of the throat?

It may be that the time will never come when doctors on occasions will not disagree, but it certainly never will come when simply the most thorough and complete anatomical knowledge will alone equip a man to take a conspicuous place as a successful physician or surgeon.

